# Advanced Pathogenetic Concepts in T-Cell Prolymphocytic Leukemia and Their Translational Impact

**DOI:** 10.3389/fonc.2021.775363

**Published:** 2021-11-19

**Authors:** Till Braun, Annika Dechow, Gregor Friedrich, Michael Seifert, Johanna Stachelscheid, Marco Herling

**Affiliations:** ^1^ Department I of Internal Medicine, Center for Integrated Oncology (CIO), Aachen-Bonn-Cologne-Duesseldorf, Excellence Cluster for Cellular Stress Response and Aging-Associated Diseases (CECAD), Center for Molecular Medicine Cologne (CMMC), University of Cologne (UoC), Cologne, Germany; ^2^ Department of Hematology and Cellular Therapy, University of Leipzig, Leipzig, Germany; ^3^ Institute for Medical Informatics and Biometry (IMB), Carl Gustav Carus Faculty of Medicine, Technische Universität Dresden, Dresden, Germany

**Keywords:** T-PLL, clonal evolution, pathogenesis, TCL1A, ATM

## Abstract

T-cell prolymphocytic leukemia (T-PLL) is the most common mature T-cell leukemia. It is a typically aggressively growing and chemotherapy-resistant malignancy with a poor prognosis. T-PLL cells resemble activated, post-thymic T-lymphocytes with memory-type effector functions. Constitutive transcriptional activation of genes of the T-cell leukemia 1 (TCL1) family based on genomic inversions/translocations is recognized as a key event in T-PLL’s pathogenesis. TCL1’s multiple effector pathways include the enhancement of T-cell receptor (TCR) signals. New molecular dependencies around responses to DNA damage, including repair and apoptosis regulation, as well as alterations of cytokine and non-TCR activation signaling were identified as perturbed hallmark pathways within the past years. We currently witness these vulnerabilities to be interrogated in first pre-clinical concepts and initial clinical testing in relapsed/refractory T-PLL patients. We summarize here the current knowledge on the molecular understanding of T-PLL’s pathobiology and critically assess the true translational progress around this to help appraisal by caregivers and patients. Overall, the contemporary concepts on T-PLL’s pathobiology are condensed in a comprehensive mechanistic disease model and promising interventional strategies derived from it are highlighted.

## Introduction

T-cell prolymphocytic leukemia (T-PLL) is an aggressive peripheral T-cell malignancy (1) and represents the most common mature T-cell leukemia in Western countries (incidence ≈ 2.0/million/year) (2). Patients suffering from T-PLL typically present with exponentially rising white blood cell counts, (hepato-) splenomegaly, and small-node lymphadenopathy. CNS involvement has been described as a severe clinical manifestation in a minority of T-PLL (<5% of cases) (3, 4). The rapidly expanding and chemotherapy-refractory course is reflected by a median overall survival from diagnosis of less than 3 years (5, 6). Up to now, the humanized CD52-antibody alemtuzumab is the only substance that induces acceptably high response rates, (in >80% of patients at first line). Notably, nearly all patients relapse within 2 years after alemtuzumab, with very limited options to salvage (4, 7).

First described in 1973 (8), the diagnosis of T-PLL was mainly based on cytomorphological characteristics (6). In the following decades, the pathogenetic concept of T-PLL was centered around cytogenetic abnormalities. *Inversions* or *translocations* of the *TCL1A* locus are the most common chromosomal aberrations and are central in establishing the diagnosis of T-PLL (9). Within the last 5-7 years, genomic and epigenomic studies have remarkably expanded our pathogenetic understanding of T-PLL. More recently, molecular hallmarks around perturbed responses to DNA damage, including repair and apoptosis, as well as alterations of cytokine signaling and epigenetic deregulations, were identified as exploitable dependencies. Here, we condense these novel advances in a comprehensive mechanistic disease concept and highlight promising interventional strategies that are being derived from it.

## Cell of Origin Concepts

In >95% of T-PLL, aberrant constitutive expression of the proto-oncogenes *TCL1A* or *MTCP1* by *inversions* or *translocations* are observed that juxtapose the *TCL1A* (at 14q32.1) or *MTCP1* (at Xq28) loci to the 14q11.2 locus and by that under control of highly active *TRA* gene enhancer elements. This prevents physiological downregulation of *TCL1A* or *MTCP*1 and is considered the initial event of T-PLL’s leukemogenesis (10). Both oncogenes have shown their oncogenic potential in transgenic mouse models (11–13). Under physiological conditions, expression of the *TCL1A* oncogene is silenced in CD4/CD8 double-positive (dp) thymocytes (14, 15). At this stage, rearrangements of the *TRA* locus, encoding for the T-cell receptor (TCR) α-chain, take place (16). Whole-genome sequencing and breakpoint analyses identified that all T-PLL had a breakpoint involving recombination signal sequences (RSS) of the J region of the *TRA* locus. On the opposite side of the *inversion/translocation*, breakpoints were more variable, but also involved classical or cryptic RSS (17). In accordance with the finding that virtually all T-PLL express the surface TCR complex (18), the other allele of the analyzed T-PLL cases showed legitimate *TRA* rearrangements, leading to the expression of a functional TCR (17). Together, these findings suggest, that the aberrant *TRA-TCL1A/MTCP1* rearrangements occur during the opening of the *TRA* locus at the CD4/CD8 dp thymocyte stage in a RAG1/2 dependent manner (17), followed by legitimate recombination of the locus on the other allele. High TCL1A expression is associated with genomic instability (19), thereby forming the basis for additional genomic hits driving oncogenesis (9, 10). However, whether the illegitimate rearrangement is the first hit in the pathogenesis of T-PLL is uncertain. A preceding mono-allelic deletion or mutation of *ATM*, which are highly recurrent in T-PLL cells, is possible as well. This is supported by a high incidence of T-PLL in patients with germline *ATM* defects as well as its involvement in the regulation of monoallelic cleavage and genomic stability during *TRA* recombination (20).

## Structural Genomic Aberrations

Complex karyotypes (≥3 structural or numerical cytogenetic aberrations) are seen in ~70% of T-PLL and were associated with a poorer prognosis (21). T-PLL genomes usually show complex somatic DNA copy number alterations (CNA) in array-based profiling (10, 21, 22). Generally, losses of chromosomal regions are more frequent than gains. These somatic CNA usually affect hundreds of genes in a patient and are not closely associated with altered expression of the respective genes, indicating additional modes of transcriptional dysregulation beyond CNA. Besides the above-described aberrations affecting genes of the *TCL1* family, genomic losses of chromosome 11q and gains of chromosome 8q are most recurrently observed. Losses affecting chromosome 11 involve the tumor suppressor *ATM* (11q22.3) as the minimally deleted region (6, 10, 19, 21–30). This is implicated in T-PLL development by dysregulation of proper DNA damage repair as highlighted by more complex karyotypes in *ATM* deleted cases (10). The genomic region encoding for the downstream effector of ATM, p53, is only disrupted in a minority of T-PLL (10). Gains of chromosome 8q can mainly be attributed to a trisomy of 8q, resulting from isochromosomes (8)(q10) (29). Overexpression of the proto-oncogene *MYC* (8q24.21) is not strictly associated with the presence of 8q gains and vice versa. Other genes like *AGO2* at 8q24.3 are more frequently involved in these 8q amplifications. Overexpression of AGO2, which centrally regulates RNA interference, may additionally contribute to T-PLL development (10).

At lower frequencies, genomic losses of chromosomes 6q, 8p, 12p, 13q, and 22q as well as genomic amplifications of 6p and 22q are observed in T-PLL cells (10, 21–23, 27). Up to now, the underlying target genes of these structural aberrations and their functional contributions have not been fully revealed. First promising concepts could derive from a systems biology approach (31). Genome-wide gene expression and copy number profiles of T-PLL patients could be utilized to learn a T-PLL specific gene regulatory network (32). Such a network would allow to predict potential impacts of individual CNA on known cellular signaling pathways or treatment response signatures by network propagation (32), as demonstrated for oligodendrogliomas (33) and prostate carcinomas (34). Thus, more intensified efforts on integrating available genome-wide data could help to identify new potential driver candidates and their downstream targets in T-PLL.

## The Mutational Profile of T-PLL

Besides the highly prevalent structural lesions involving the oncogenes *TCL1A*, *AGO2*, and *MYC*, as well as in the tumor suppressor *ATM*, various single-nucleotide variants (SNVs) were linked to the molecular pathogenesis of T-PLL cells (10, 26, 35, 36). Generally, SNVs occur at similar rates in T-PLL as in other hematologic and solid tumors (10). Most of these primarily somatic SNVs seem to accumulate during T-PLL’s leukemogenesis in the context of high levels of oxidative damage and in the absence of efficient repair mechanisms to counteract these hazards (10). Fittingly, *ATM*, the central apical regulator of DNA integrity, shows high rates of damaging SNVs, in addition to the above-described partial inactivation by mono-allelic losses (10, 24, 26, 35–38). These missense, nonsense, or frameshift mutations of *ATM* mainly cluster within its FAT or PI3K domains (10).

Other frequently mutated genes in T-PLL are *CHEK2*, *SAMHD1*, and *MSH*, which are also involved in DNA damage repair mechanisms, which further supports a concept of T-PLL’s incompetence in safeguarding mechanisms of repair or cell death execution (10, 26, 35, 36). Remarkably, *SAMHD1* and *ATM* belong to the small fraction of genes, whose mutations show variant allele fractions (VAFs) of more than 80% (10, 35), suggesting acquisition of these lesions early in leukemogenesis.

Within the last decade, genomic aberrations affecting the JAK/STAT signaling pathway emerged as an additional hallmark of T-PLL (10, 26, 35, 36, 38–42). The *JAK3* gene shows the highest frequency of such gain-of-function mutations, followed by *STAT5* and *JAK1 (* 43). These primarily missense mutations target the conserved pseudokinase (*JAK1*, *JAK3*) or SH2 domains (*STAT5*) in most T-PLL cases. Notably, SNVs affecting components of the JAK/STAT signaling pathway occur at relatively low VAFs, indicating their rather sub-clonal character (10). However, the central role of deregulated JAK/STAT signaling is substantiated by genomic losses of genes that encode for negative regulators of this pathway (e.g. *DUSP4*, *SOCS* genes) (43). Together with the high frequency of *JAK*/*STAT* gene mutations, basal phosphorylation of distal STAT5 is observed in virtually every T-PLL case (10, 43). In addition, the WNT as well as the Notch signaling pathways, are disturbed by SNVs in a minority of T-PLL cases (10, 26). Rare mutations further involve cell cycle regulation (e.g. *CDC27*) and apoptosis regulation (e.g. *BCLAF1*) *(* 10).

## The Transcriptomic Landscape

Analyses of the transcriptome of T-PLL cells have been performed intensively in bulk RNA samples, either by gene expression arrays or by RNA sequencing (RNA-seq). In line with rearrangements of the chromosome 14q, *TCL1A* was the most upregulated gene in virtually every cohort (10, 35, 42, 44). The other TCL1 family members, *TCL1B* and *MTCP1*, showed additional overexpression, although to a lower extent (10). In agreement with the gains at chromosome 8q, the proto-oncogene *MYC* as well as the miR-processing regulator *AGO2* showed overexpression on mRNA level (10, 42). Highlighting the importance of deregulated JAK/STAT signaling in T-PLL, downstream targets of this pathway (e.g. BCL2L1) showed a significant upregulation (42).

Among the genes with the most significantly altered expression were those involved in TCR/cytokine signaling. Prominent examples are downregulated *CTLA4* and *SLAMF6*. They are central mediators of immune signal transduction and regulation of lymphocyte activation and we implicate their loss in the activated T-cell phenotype of the T-PLL cell (10, 18, 22). Moreover, potential underlying causes for the inability of T-PLL cells to undergo cell death upon DNA damage were identified in their altered transcriptome: Pro-apoptotic genes (e.g. *GIMAP5*, various Caspases) were significantly downregulated (10, 22). Transcriptome studies can also be utilized to identify individualized treatment options for T-PLL patients. In a first case study, RNA-seq data were integrated with exome-seq and *ex vivo* single-drug sensitivities, establishing a customized platform on individual predictions of responses to drug combinations (39).

## The miR-ome of T-PLL Cells

Recently, the miR-ome of T-PLL cells was analyzed by small RNA-seq in two independent cohorts (44, 45). T-PLL cells showed a global miR expression signature of ~35 significantly deregulated miRs, resembling the miR expression profile of TCR-activated healthy T-cells (45). By combining the small RNA-seq with transcriptome sequencing data, regulatory networks involving cell survival signaling and DNA repair pathways were uncovered. In both cohorts, the miR-141/200c cluster showed the strongest upregulation among all miRs and separated T-PLL cases into two major subgroups with normal vs. upregulated expression. Preliminary data revealed a role of this cluster in TGF-β signaling (44) as well as in cell cycle regulation (45). Further perturbations of miR expression include overexpression of miR-223-3p and miR-181a/miR-181 as well as downregulation of the miR-21 and the miR-29 cluster. The functional consequences of these deregulations have yet to be demonstrated in T-PLL. Nevertheless, based on the expression of miR-200a-3p, miR-223-3p, and miR-424-5p, a first overall survival score for T-PLL (miROS-TPLL) was established and might improve clinical stratifications (45).

## Epigenetic Alterations

Gene set enrichment analyses of T-PLL transcriptomes identified pathways of epigenetic regulation as significantly altered (10). These findings were additionally highlighted by a high incidence of mutations in epigenetic modifiers (e.g. *EZH2*, *TET2*, *KMTs*) *(* 10, 26, 35, 36). However, systematic analyses of DNA-methylation, profiles of histone modifications, and states of chromatin accessibility have not yet been published. First data in a small cohort of T-PLL implicate massive epigenetic reprogramming, as shown by genome-wide alterations of chromatin states at promoters and active enhancers identified *via* H3K4me3 and H3K27ac ChIP-seq (46). These alterations correlated with changes in expression of frequently deregulated genes (e.g. *TCL1A*, *MYC*, *EZH2*, *AGO2*), presenting additional ways of their deregulation beyond the described genomic aberrations. Vice versa, a role of *TCL1A/MTCP1* activation and/or *ATM* inactivation in epigenetic disturbances is also conceivable (47, 48).

## The Microenvironment of T-PLL Cells

Besides (epi)genetic changes, the dependence of leukemic cells on signals from microenvironmental sources for proliferation and survival has been shown for various entities, including T-cell neoplasms (49). Such interactions are mediated by adhesion molecules, cell surface ligands, chemokines, cytokines, and their respective receptors (50). So far, little is known about the (specific) micromilieu of T-PLL cells and how they shape it. Upregulation of cytokines (e.g. TNF, IL-8), cytokine receptors (e.g. CD25 (IL-2Rα), CD122 (IL-2Rβ), CD124, or CD127), as well as of chemokine receptors (e.g. CCR3 and CCR4) provide first hints of a deregulated crosstalk between T-PLL and bystander cells (18). Furthermore, mutations of chemokine receptors (e.g. CXCR3) are described (10). The potential proactive role of the micromilieu in T-PLL’s leukemogenesis is further implicated by the secretion of the Th1-associated cytokines IFN-γ, IL-2, IL-10, TNF-α/β, and IL-8 of T-PLL cells upon TCR stimulation (18). Mechanistic proof for an involvement of CCR7 in the sustenance of T-PLL cell survival derives from studies with CCR7-blocking antibodies. They impaired survival signaling pathways in T-PLL cells *in vitro* and increased the survival of mice transplanted with the T-PLL-like cell line SUP-T11 (51). More work is required to study the composition of T-PLL’s microenvironment (i.e. cell types and humoral factors) and the involved molecular interactions.

## Role of the T-Cell Receptor

TCR signaling is the major growth regulatory system of T-cells. It shapes their maturation, differentiation, and activation, hence their effector and tolerogenic capacity (52, 53). Amplification of TCR signaling represents a feature of many T-cell malignancies, although generated by distinct mechanisms (54): (*i*) decreased input thresholds for continuous exogeneous TCR activation, (*ii*) autonomous activation of TCR-signaling intermediates, (*iii*) downregulation of inhibitory coregulators, or (*iv*) stand-ins for TCR signals, such as strong cytokine-inputs or their mimics, e.g. *via* the ALK oncogene. T-PLL cells usually express at least one surface component of the TCR/coreceptor complex and show robust TCR-signal competence when stimulated *ex vivo (* 9, 18). Their gene expression profiles show prominent signatures of TCR activation (10). Notably, TCL1A acts as a physically engaging coactivator of TCR-kinases such as AKT, ZAP70, or ERK, and by that is a TCR-signal enhancer, hence, a sensitizer towards low-abundance signals. That places T-PLL into model (*i*) of the TCR-centric pathogenetic view of T-cell neoplasms (18, 54).

Enhanced TCR signaling is further established in T-PLL cells by impaired control mechanisms [model (*iii*)], e.g. by downregulation of negative coregulators such as SLAMFs or checkpoint molecules such as CTLA4 (10). The resulting activated phenotype of T-PLL cells is additionally accompanied by a TCL1A-mediated inability to execute FAS-mediated and activation-induced cell death (18).

In line with their TCR signaling competence, T-PLL cells reveal a phenotype of mature, antigen-experienced, non-conventional memory T-cells (18). As an underlying principle, it is tempting to speculate that through enhanced TCR signaling, the transition of naïve T-cells into an expanding pool of memory T-cells is accelerated. The lack of a common TCR clonotype across cases would indicate that not a specific antigen drives TCR-mediated outgrowth in T-PLL (18, 55). More likely is an MHC-dependent TCR activation through various low-avidity (auto)antigens or antigen-independent tonic signals at place, either MHC-driven or *via* TCR self-activation in enabled memory T-cells. Although treatment strategies that target TCR signaling intermediates have shown promising potential (56), the TCR dependence of T-PLL cells at the overt leukemic stage is not conclusively clarified.

## Discussion

### Model of Clonal Evolution of T-PLL Cells

**Figure 1:**
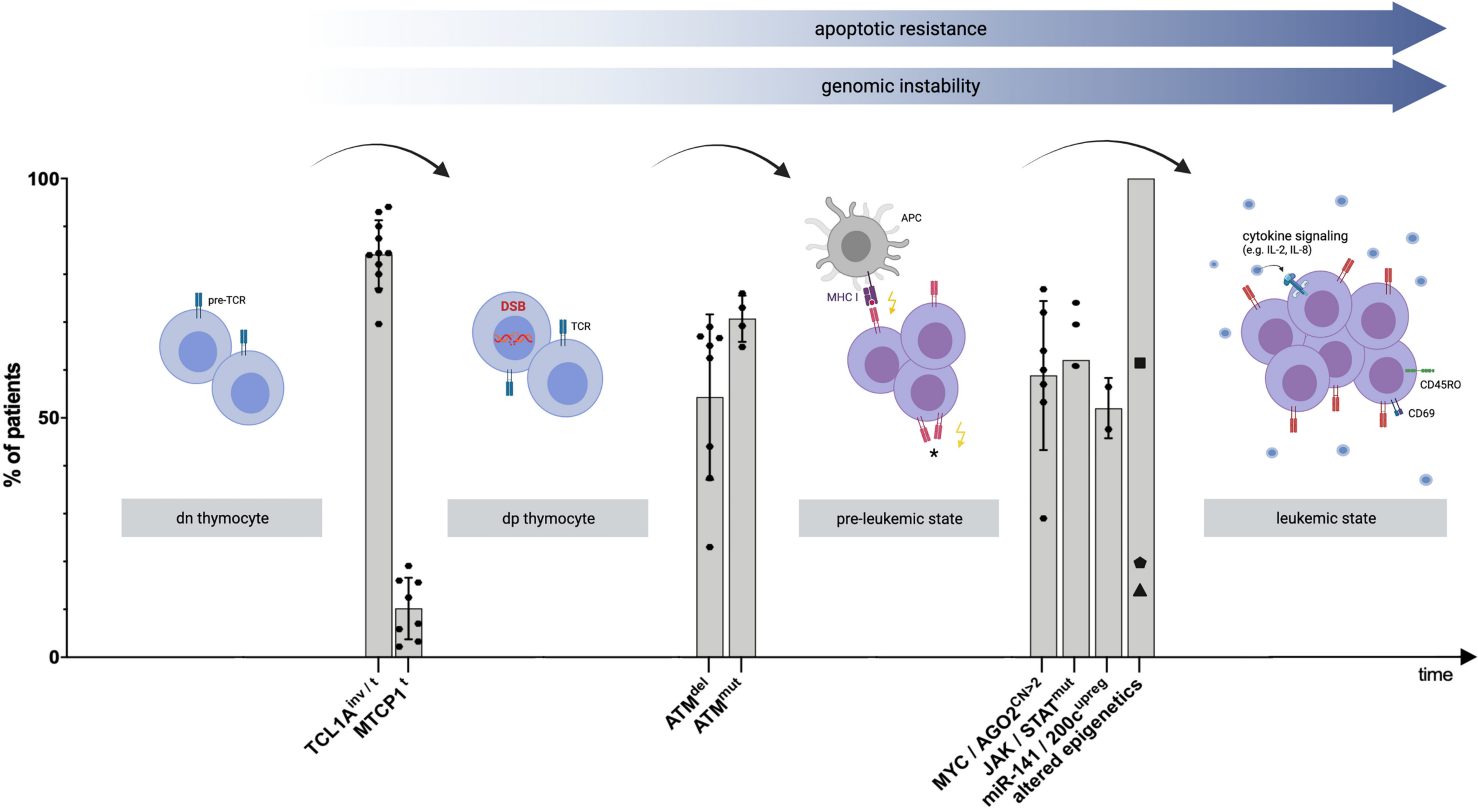
Proposed model of clonal evolution of T-PLL cells. Schematic visualization explaining T-PLL’s leukemogenesis, based on recent genomic profiling series and corresponding functional assessments. Timeline: Chronology of genomic events leading to the progression to an advanced state of (pre)malignant T-cell development. Y-Axis: Percentage of all analyzed T-PLL patients presenting the respective genomic aberration. Each dot represents a prevalence, derived from selected publications (**Supplementary Table 1**). The median, as well as standard deviation, out of these publications was calculated for each genomic event. The variability between the studies can be attributed to the different methods and cohort sizes (for more information refer to **Supplementary Table 1**). The first ‘stage’ involves the double-negative (dn) thymocyte, carrying the pre-T cell receptor (pre-TCR) complex. *Translocations* (*t*) and *inversions* (*inv*) of chromosome 14q at the dp thymocyte stage result in constitutive expression of the proto-oncogenes *TCL1A* or *MTCP1* in a vast majority of T-PLL cases (9, 17). These hits impair the genomic stability of the affected T-cell by reduced DNA repair capacities of DNA double-stranded breaks (DSB) or other (oxidative) insults (10). *Deletions* (*del*) and *mutations* (*mut*) involving ATM lead to a functionally hypomorphic apical regulator of repair, cell fate, and cell cycle control of the T-PLL precursor. This pre-leukemic cell becomes unable to execute such safeguarding mechanisms upon genotoxic stress (10). Among subsequent perturbations, TCL1A overexpression lowers TCR-signaling thresholds (18), enabling the cell to sustain on low-level input, either by major histocompatibility complex (MHC)-dependent (auto) antigen-presenting cells (APC), or by self-MHC drive only, or by autonomous TCR activation (*, not proven). A central distal node is the JAK/STAT transcriptional machinery. Besides major growth pathways such as the TCR and cytokine-mediated cascades feeding into it, there also is a high prevalence of hyperactivating mutations that target *JAK1*, *JAK3*, or *STAT5B (* 18) and a high incidence of losses of JAK/STAT negative regulators (43). Further leukemic outgrowth and progression to an exponentially proliferating T-PLL cell are likely mediated by additional aberrations, including copy number (CN) gains on chromosome 8q, leading to *MYC* amplification and *AGO2* overexpression (10). Furthermore, deregulations of T-PLL’s miR-ome, exemplarily represented by the upregulation (upreg) of the miR-141/200c family (45), and of T-PLL’s epigenome in virtually all patients as shown by altered chromatin states at promoters and active enhancers (46), potentially mediated by frequent mutations in *KMTs*(▪), *TET2* (⬟), and *EZH2* (▲) (10), contribute to the final leukemic outgrowth of a transformed and activated T-cell (as shown by the T-cell activation marker CD69) with memory-type effector functions (as shown by CD45RO surface expression) (18). The figure was created by the authors using Biorender.com.

Recent advances in omics technologies over the last decade have elevated the molecular understanding of T-PLL to another level (**Figure 1**, **Supplementary Table 1**). *Translocations* and *inversions* of chromosome 14q at the dp thymocyte stage are perceived to initiate T-PLL’s leukemogenesis (10, 17). These genomic aberrations lead to overexpression of the proto-oncogenes *TCL1A* and *MTCP1* and result in apoptotic resistance and genomic instability (19). TCL1 family-activating lesions form a functionally perturbing cooperation with (preceding or subsequent) lesions that impair the tumor suppressor ATM, which further incapacitate the T-PLL cell to execute safeguarding responses (10). Likely, additional perturbations are operational for this TCL1^up^/ATM^def^ leukemic precursor to finally escape T-cell homeostatic control. These are acquired by lesions that activate JAK/STAT signaling (43), by miR (processing) deregulations (44, 45), by *MYC* amplification (6, 10), and by deregulated epigenetic mechanisms (10, 36). To a lesser degree we understand, on which central functional levels, such as TCR- or cytokine signaling or autocrine forward-feeding loops, these (epi)genetic events have a direct or less immediate impact.

Overall, many questions of T-PLL’s pathogenesis remain unresolved, like (*i*) the role of pro-survival signals of T-PLL’s bystander cells, (*ii*) the dependence of T-PLL cells on their TCR in clonal sustenance, (*iii*) the nature of T-PLL’s epigenome, and (*iv*) the mechanisms of disease progression and treatment resistance. Especially the latter aspect calls for single-cell resolved analyses to illustrate clonal oscillations.

### Clinical Implications Derived From the Current Disease Model

The identification of key drivers of the molecular pathogenesis of T-PLL offers the possibility for the development of new drugs that target its crucial pathways. Here, central pathogenetic relevance is likely not equivalent to a major vulnerability, which requires more thorough interrogations. However, there is sound reason to be optimistic that we will soon see novel strategies against T-PLL cells to become the basis for future combinatorial therapies. Exemplarily, agents targeting TCR signaling or the JAK/STAT pathway (18, 56) show encouraging results, preclinically and/or in first case reports (57, 58). In addition, the inability of T-PLL cells to induce adequate responses to DNA insults was translated into therapeutic strategies to reactivate p53 *via* MDM2/MDMx inhibitors or targeting BCL2 family members (e.g. Venetoclax) (10, 59, 60). There are ongoing activities in the search for efficacious combinations of the, as single agent clinically only moderately active Venetoclax, with other classes of inhibitors in relapsed/refractory (r/r) T-PLL (59–62). In addition, epigenetic disturbances of T-PLL cells further emphasize hypomethylating agents (e.g. Cladribine) as well as inhibitors of deacetylating enzymes (e.g. Romidepsin) as options (10, 63, 64). Combining these drugs, which target molecular vulnerabilities of T-PLL cells, with the current standard therapy of alemtuzumab represents another promising approach. Another challenge to be addressed is the ‘purposing’ of the innate or adaptive immune system to specifically attack T-PLL cells (65).

## Author Contributions

TB, AD, GF, MS, JS, and MH contributed to initial and subsequent drafts of the manuscript. TB, AD, and MH designed and drew the figure and corresponding table. All authors contributed to the article and approved the submitted version.

## Funding

This research was funded by the DFG Research Unit FOR1961 (Control-T; HE3553/4-2), the Köln Fortune Program, and the Fritz Thyssen Foundation (10.15.2.034MN). This work was also funded by the EU Transcan-2 consortium ‘ERANET-PLL’ (01KT1906A/B) and by the ERAPerMed consortium ‘JAKSTAT-TARGET’ (ERAPERMED2018-066).

## Conflict of Interest

The authors declare that the research was conducted in the absence of any commercial or financial relationships that could be construed as a potential conflict of interest.

## Publisher’s Note

All claims expressed in this article are solely those of the authors and do not necessarily represent those of their affiliated organizations, or those of the publisher, the editors and the reviewers. Any product that may be evaluated in this article, or claim that may be made by its manufacturer, is not guaranteed or endorsed by the publisher.
